# Opposing shifts in distributions of chlorophyll concentration and composition in grassland under warming

**DOI:** 10.1038/s41598-021-95281-3

**Published:** 2021-08-03

**Authors:** Yao Zhang, Nianpeng He, Guirui Yu

**Affiliations:** 1 Key Laboratory of Ecosystem Network Observation and Modeling, Institute of Geographic Sciences and Natural Resources Research, Chinese Academy of Sciences, 11A, Datun Road, Chaoyang District, Beijing, 100101 China; 2 College of Resources and Environment, University of Chinese Academy of Sciences, Beijing, 100049 China; 3 Key Laboratory of Vegetation Ecology, Ministry of Education, Institute of Grassland Science, Northeast Normal University, Changchun, 130024 China

**Keywords:** Plant sciences, Ecology

## Abstract

Global warming has significantly altered the distribution and productivity of vegetation owing to shifts in plant functional traits. However, chlorophyll adaptations—good representative of plant production—in grasslands have not been investigated on a large scale, hindering ecological predictions of climate change. Three grassland transects with a natural temperature gradient were designed in the Tibetan, Mongolian, and Loess Plateau to describe the changes in chlorophyll under different warming scenarios for 475 species. In the three plateaus, variations and distributions of species chlorophyll concentration and composition were compared. The results showed that the means of chlorophyll concentration and composition (chlorophyll a/*b*) increased with the mean annual temperature. Still, their distributions shifted in opposite manners: chlorophyll concentration was distributed in a broader but more differential manner, while chlorophyll composition was distributed in a narrower but more uniform manner. Compared to chlorophyll concentration, chlorophyll composition was more conservative, with a slight shift in distribution. At the regional level, the chlorophyll concentration and composition depend on the limitations of the local climate or resources. The results implied that warming might drive shifts in grassland chlorophyll distribution mainly by alternations in species composition. Large-scale chlorophyll investigations will be useful for developing prediction techniques.

## Introduction

Ongoing climate change has influenced vegetation in several ways by affecting plant growth, distribution, adaptive strategy, and productivity^1^. One significant effect of global warming is the migration of the distribution boundaries of individual plant species and alpine tree lines towards high latitudes^2^. It is believed that temperature change affects the production capacity and adaptive strategies of plants mainly by altering their functional traits^3^.

Chlorophyll (Chl), which harvests, transfers, and converts sunlight, is the initiator of photosynthesis and is thus acknowledged as the best indicator of productivity^4^. The fluorescence of these tiny pigment molecules allows their large-scale detection and is, therefore, a promising tool for estimating productivity and undertaking ecological predictions^5^. Terrestrial plants possess two types of Chl: Chl *a* and Chl *b*. Both have strong light absorption capacities; the concentrations of Chl (Chl *a*, Chl *b*, and Chl *a* + *b*) are indicators of light-use efficiency and, in turn, production efficiency. In addition, Chl *a* and Chl *b* are the main components of the reaction centre (RC) and light-harvesting complexes (LHC) in the photosystem (PS). Hence, the composition of Chl (i.e., Chl *a*/*b*) mainly reflects the allocation of plants to different photosynthetic apparatus protein complexes^6^ as well as the efficiency balance between light-harvesting and electron transport^7^.

Leaf Chl concentration (Chl *a*, Chl *b*, and Chl *a* + *b*) and composition (Chl *a*/*b*) may be influenced by various environmental factors. Light is one of the primary factors that induce Chl synthesis^8^. Some reports have concluded that Chl concentration and light availability are controversial and depend on taxonomy^9,10^ or functional groups^11^. In addition, Chl adaptation mechanisms involve functional coordination among different levels of the organism, physiologically and morphologically^9,12,13^. Along high to low light gradients, Chl *b* increases, while Chl *a*/*b* decreases to enhance the utilisation of diffused radiation^9,14^. However, these conclusions have been drawn based on studies of forests or woody plants in areas with large light gradients between the canopy and understory^14,15^. Apart from light, Chl synthesis may also be influenced by temperature, water, and N availability. However, Chl concentration and composition adaptation to these environmental factors has not been revealed in grasslands on a large scale.

Distributions of functional traits may determine community assembly and function under environmental change because the value range of a functional trait indicates adaptation to stressful ends of environmental gradients^16–18^. The trait distribution can be demonstrated using a frequency curve with a shape described mainly by skewness and kurtosis. A skewed distribution with high kurtosis indicates that most species share similar trait values; hence, trait convergence can be identified. Theoretically, traits within a community will converge under environmental filtering^19^ because stressful environments can force the trait to deviate from the original distribution with characteristics of large skewness and kurtosis^20,21^. Thus, among communities that cross large environmental gradients, there should theoretically be trait divergence. However, an increasing number of studies have argued that traits diverge more within communities than among communities across environmental gradients, with functionally contrasting species co-occurring within communities^22–24^. Large inter- or intra-species variation has great significance in sustaining the multi-functionality of communities in stressful environments^17^. However, Chl distribution and variation on large scales under global warming are not yet clear, which undoubtedly limits the technological innovation of prediction.

**Figure 1:**
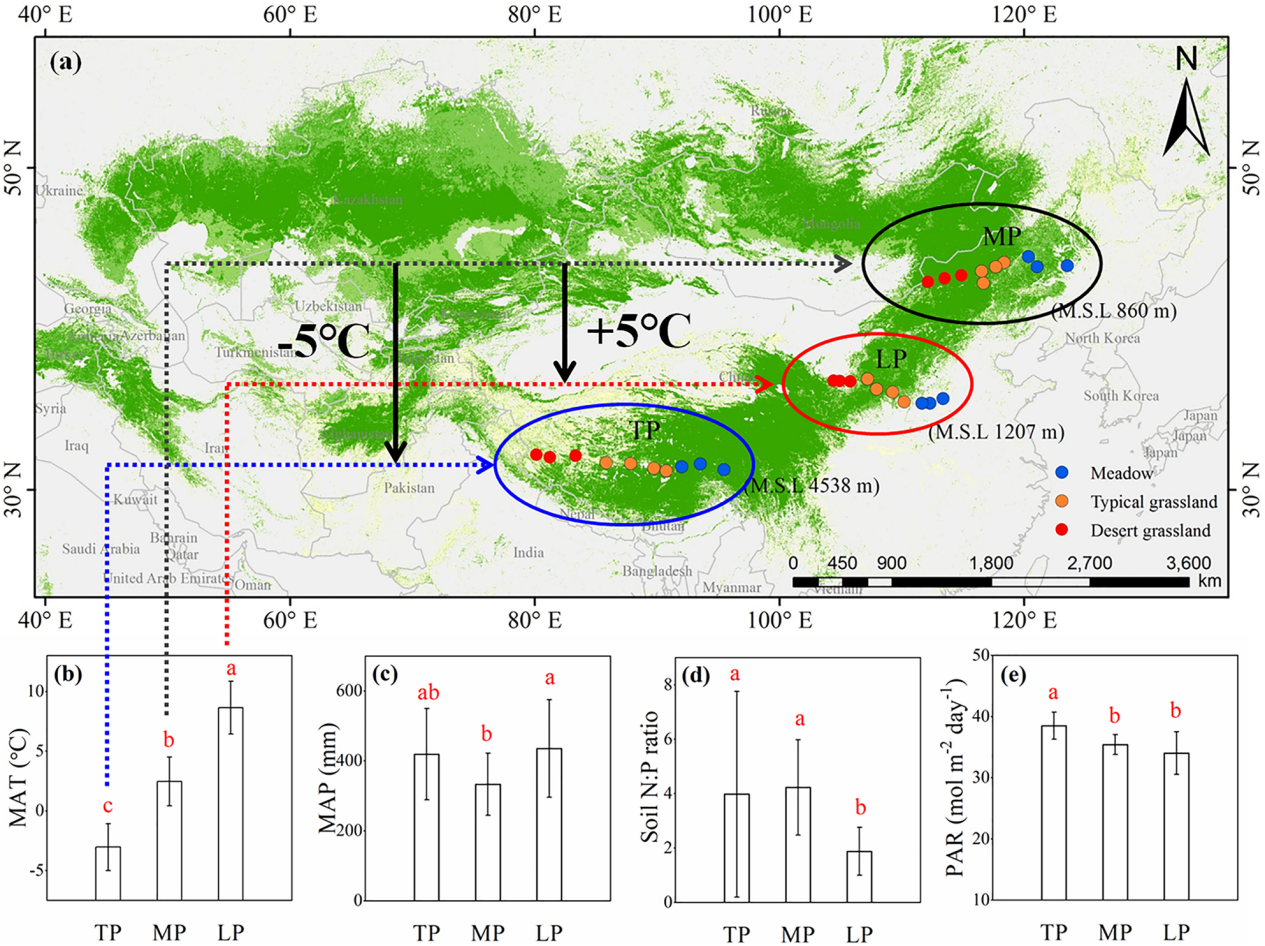
Spatial location of sites in three plateaus transects of northern hemispheric grassland. The mean annual temperature (MAT) differences of ± 5 °C were considered from lowest in TP, middle in MP, to highest in LP. Three grassland types were involved in each plateau: meadow, typical grassland and desert grassland from east to west (**a**). At the regional levels, the primary limiting factors for plant growth in TP, MP and LP were MAT (**b**), mean annual precipitation (MAP; **c**), and soil nutrient (soil N:P ratio; **d**), respectively. The three plateaus all received strong photosynthetically active radiation (PAR), especially TP (**e**). *TP* Tibetan Plateau, *MP* Mongolian Plateau, *LP* Loess Plateau.

In this study, three comparative grassland transects with a natural temperature gradient were designed in the Tibetan Plateau (TP), Mongolian Plateau (MP), and Loess Plateau (LP) to explore Chl adaptation to global warming (Fig. 1a). The concentration (Chl *a*, Chl *b*, and Chl *a* + *b*) and composition (Chl *a*/*b*) of Chl for all the species accessible in each plateau (a total of 475 species belonging to 264 genera of 72 families) were analysed and compared for distribution, variation, and response to major environmental factors. We hypothesised that: (1) increased temperature may lead to higher and more converged Chl concentration distributions, according to the observed north-shifting of the tree line. Still, Chl composition would be constant due to the uniform radiation of plateau grasslands; and (2) Within the three comparative plateaus, the Chl concentration and composition may be influenced primarily by their own limiting factors.

## Results

### Chlorophyll concentration of northern hemispheric grasslands

**Figure 2:**
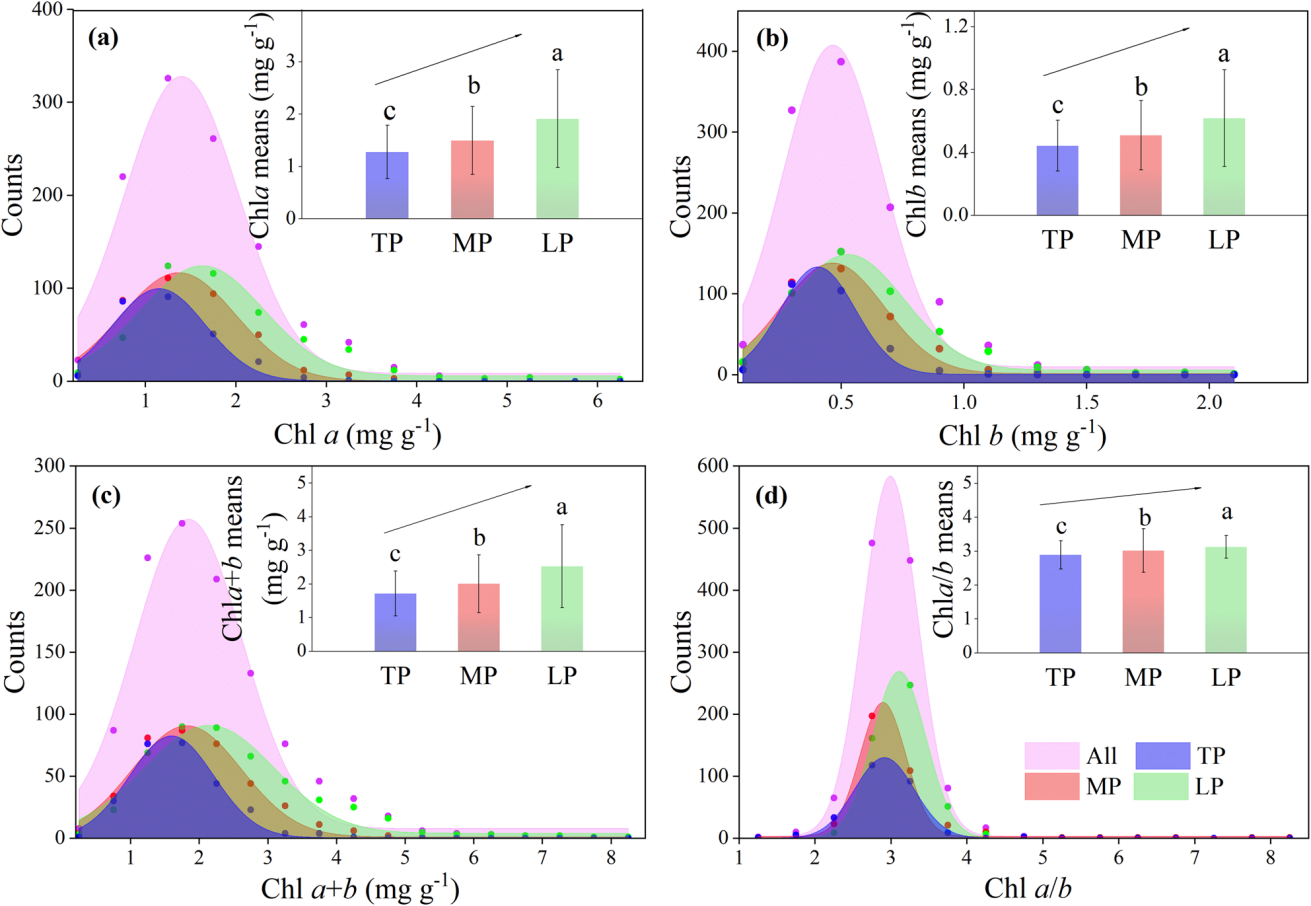
Frequency distributions of (**a**) chlorophyll *a*, (**b**) chlorophyll *b*, (**c**) chlorophyll *a* + *b*, and (**d**) chlorophyll *a*/*b* for three plateaus and the entire northern hemispheric grasslands. Averages (bar: standard deviation) were compared in the inserted figures and significances in different lowercase letters (*p* < 0.05). *Chl* chlorophyll, *TP* Tibetan Plateau, *MP* Mongolian Plateau, *LP* Loess Plateau, All: all species across the northern hemispheric grassland transects.

**Figure 3:**
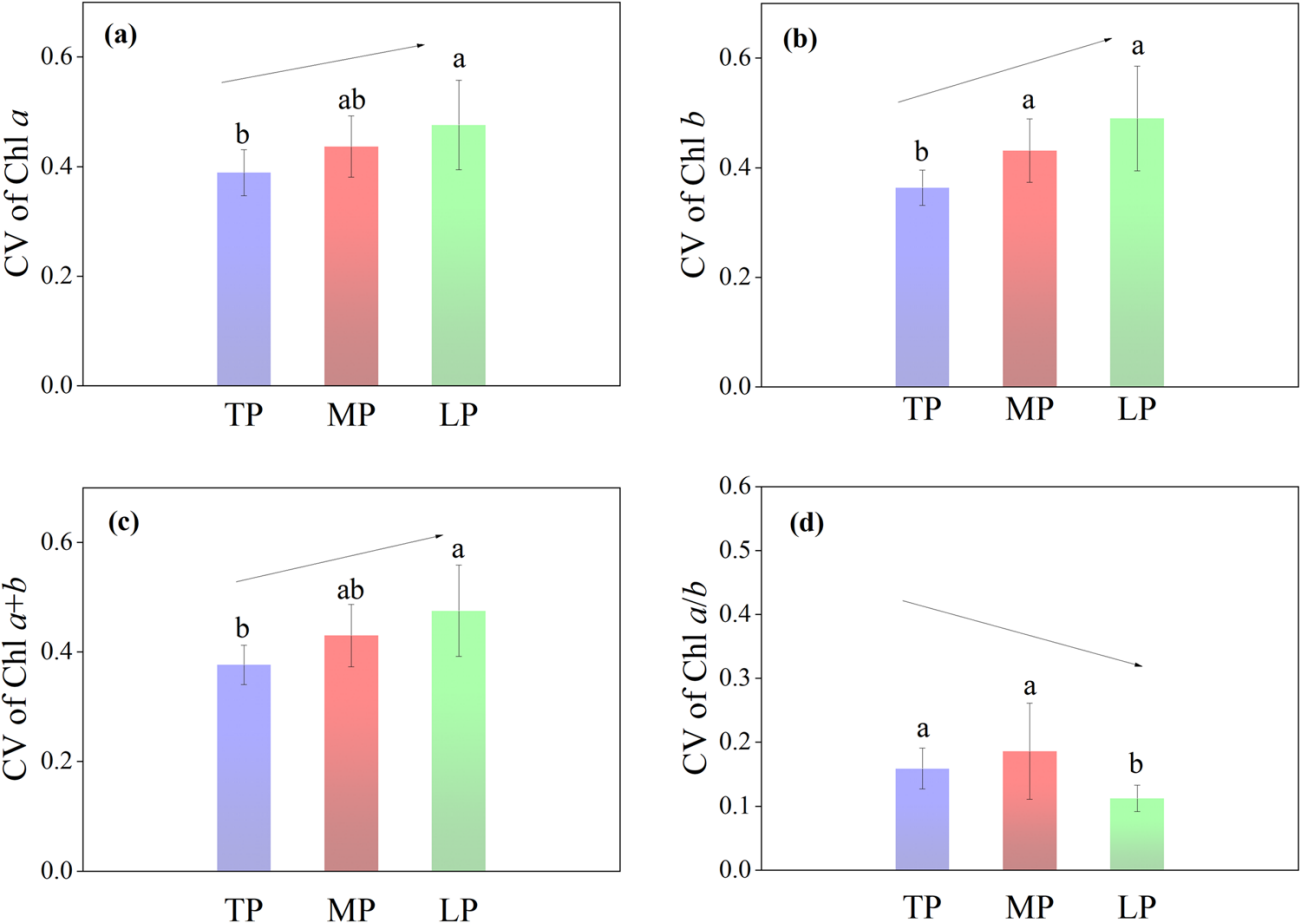
Coefficients of Variation (CV) of (**a**) chlorophyll *a*, (**b**) chlorophyll *b*, (**c**) chlorophyll *a* + *b*, and (**d**) chlorophyll *a*/*b* for three plateau grasslands. Bars denote standard deviations. Significances are in different lowercase letters (*p* < 0.05). *Chl* chlorophyll, *TP* Tibetan Plateau, *MP* Mongolian Plateau, *LP* Loess Plateau.

**Figure 4:**
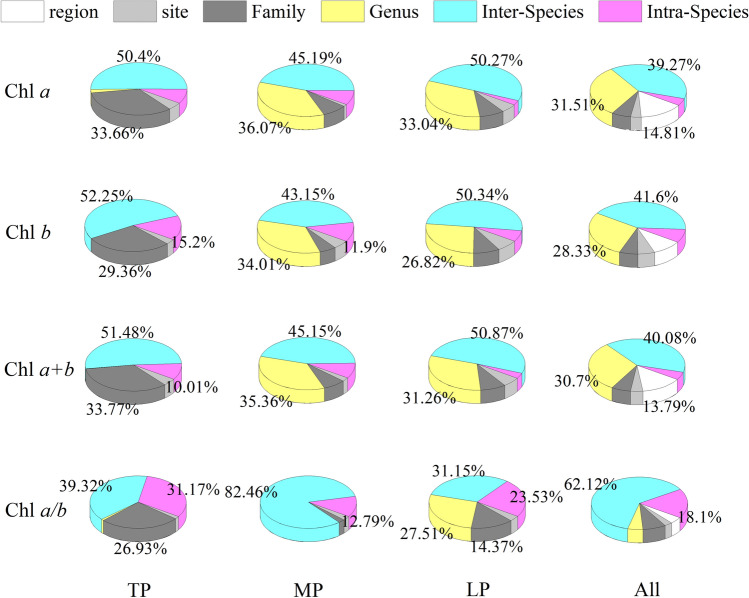
Nested analyses of variance for chlorophyll *a*, *b*, *a* + *b* and *a*/*b*. Contributing percentages of groups that were larger than 10% are given. Chl: chlorophyll, TP: Tibetan Plateau, MP: Mongolian Plateau, LP: Loess Plateau, All: all species across northern hemispheric grassland transects.

Significant positively skewed distributions for Chl *a*, Chl *b*, and Chl *a* + *b* were observed in the entire northern hemispheric grassland (Fig. 2a–c; Supplementary Table S1; Note S1; Fig. S1). In the three plateaus, the mean Chl concentrations (Chl *a*, Chl *b*, and Chl *a* + *b*) significantly increased along the ± 5 °C gradient from TP to LP (Fig. 2a–c). The coefficients of variation (CV) for Chl *a*, Chl *b,* and Chl *a* + *b* also exhibited increasing trends from TP to LP (Fig. 3a–c; Supplementary Table S1). Nested-analysis of variance (ANOVA) showed that taxonomy contributed to the majority of Chl concentration CVs rather than regions or sites. Inter-specific differences among the different classification levels contributed the most to variations in Chl concentration regionally and in the entire northern grassland (Fig. 4).

**Figure 5:**
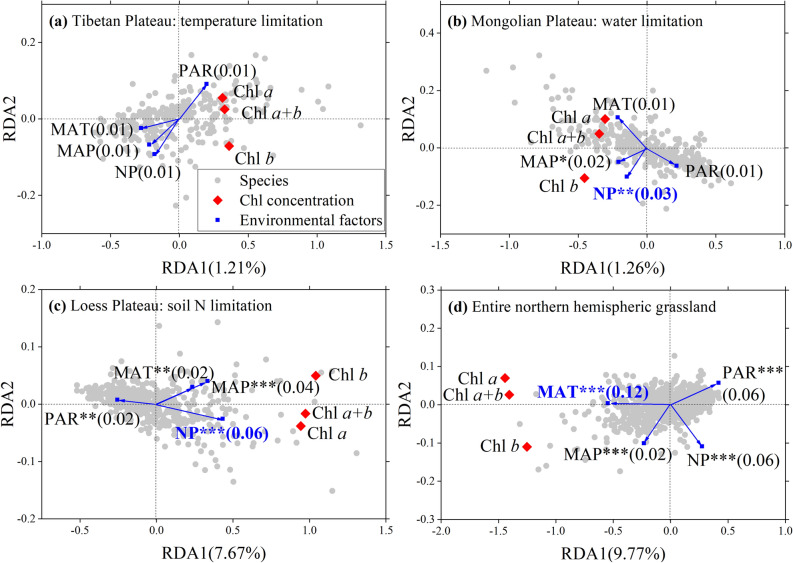
Redundancy analyses (RDA) of environmental effects (blue arrows) on chlorophyll concentrations (red diamonds) for (**a**–**c**) three plateaus and (**d**) entire northern hemispheric grassland. Chl: chlorophyll, PAR: photosynthetically active radiation, *MAP* mean annual precipitation, *MAT* mean annual temperature, *NP* the ratio of soil nitrogen to phosphorus. Significance is denoted by asterisks (*p* < 0.001***, *p* < 0.01**, *p* < 0.05*). Numbers in parentheses are the *R*^2^ values of factors, and the factors with the largest *R*^2^ values are in blue.

The concentration and composition of Chl were independent of each other, despite the region, but close positive linear relationships were exhibited among Chl *a*, Chl *b*, and Chl *a* + *b* (Supplementary Fig. S2). To remove the influences of orthogonal relationships between concentration and composition during dimension reduction analysis, redundancy analysis was performed for concentration (Chl *a*, Chl *b*, and Chl *a* + *b*) and composition (Chl *a*/*b*) separately. In TP, non-significant influences were observed for all environmental factors on Chl concentration (Fig. 5a). In MP and LP, the influencing factors that ranked first and second were soil N:P ratio and mean annual precipitation (MAP) (Fig. 5b,c). For all species in the entire northern hemispheric grassland, the first axis explained 9.77% of the total variance of Chl concentration, and the major environmental factors all exerted significant roles. Still, mean annual temperature (MAT) was the primary factor (Fig. 5d).

### Chlorophyll composition of northern hemispheric grasslands

A significant, positively skewed distribution for Chl *a*/*b* was observed in the entire northern grassland. The kurtosis of Chl *a*/*b* was far higher than that of Chl *a*, Chl *b*, and Chl *a* + *b* (Supplementary Table S1, Note S1). The average Chl *a*/*b* ratio significantly increased along the ± 5 °C gradient from TP to LP (Fig. 2d). Far smaller CVs for Chl *a*/*b* than for Chl concentration were found (Fig. 3d; Supplementary Table S1). A weak but significant reduction was found for the CVs of Chl *a*/*b* across the ± 5 °C gradient from TP to LP (Fig. 3d). Taxonomies (especially inter-specific variation) also contributed to the majority of Chl *a*/*b* variations, regionally or on the whole (Fig. 4).

For the Chl *a*/*b* of all the species, the first major axis accounted for less of the total variance than concentration, but all the primary factors exerted significant effects (Table 1). Soil N:P ratio and MAT were the most important influencing factors for Chl *a*/*b* in the entire grassland. In regions, photosynthetically active radiation (PAR), soil N:P ratio, and MAP were the primary controlling factors in TP, MP, and LP, respectively (Table 1).

**Table 1:** Redundancy analyses (RDA) of environmental effects on chlorophyll composition (Chl *a*/*b*).

	Tibetan Plateau	Mongolian Plateau	Loess Plateau	All
**Axis eigenvalues**				
Total	1.00	1.00	1.00	1.00
Constrained	0.01	0.01	0.06	0.04
Unconstrained	0.99	0.99	0.94	0.96
**Factors *R***^**2**^				
MAT	0.01	0.02*	0.25*** (−)	0.60***
MAP	0.01 (−)	0.44*** (−)	0.57*** (−)	0.10*** (−)
NP	0.08*** (−)	0.60*** (−)	0.19*** (−)	0.61*** (−)
PAR	0.12***	0.01	0.09***	0.11*** (−)

Significance is denoted by asterisks (*p* < 0.001***, *p* < 0.05). Minus signs in parentheses represent the negative effects on Chl *a*/*b*, and without this symbol are positive effects.

*MAT* mean annual temperature; *MAP* mean annual precipitation; *NP* ratio of soil nitrogen to soil phosphorus; *PAR* photosynthetically active radiation.

### Distributions of chlorophyll concentration and composition under warming

**Figure 6:**
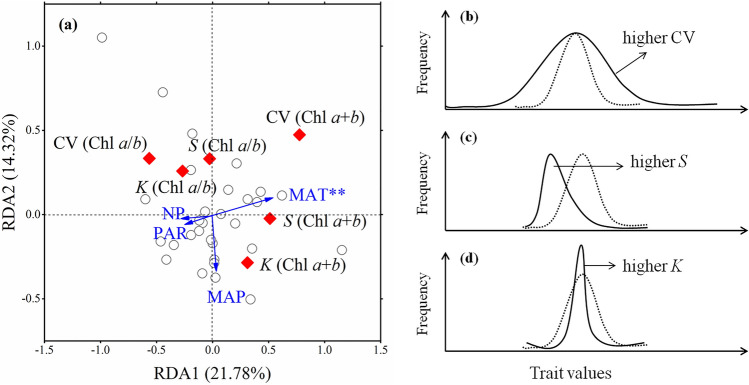
Redundancy analyses (RDA) of environmental effects on chlorophyll distribution (**a**) and sketches explaining alterations in CV (**b**), skewness (**c**) and kurtosis (**d**). The hollow circles in (**a**) represent the 30 sites of northern hemispheric grassland. Blue arrows denote environmental factors. *PAR* photosynthetically active radiation, *MAP* mean annual precipitation, *MAT* mean annual temperature, *NP* the ratio of soil nitrogen to soil phosphorus. Red diamonds denote chlorophyll distribution indicators. *Chl* chlorophyll, *CV* coefficients of variation, *S* skewness, *K* kurtosis. The asterisks denote significance (*p* < 0.01**). The black dashed curves in (**b**–**d**) represent the original or normal distributions, and the black solid lines represent the distribution shifts.

In mentioning the characteristics of the distribution, we refer to CV, skewness (*S*), and kurtosis (*K*) (Supplementary Note S1, Fig. S1). MAT exerted significant positive effects on the CV, *S*, and *K* of Chl *a*, Chl *b*, and Chl *a* + *b* distributions but weaker negative effects on CV, *S*, and *K* of Chl *a*/*b* distributions (Fig. 6a). Other environmental factors did not affect the distribution of Chl concentrations and composition.

## Discussion

**Figure 7:**
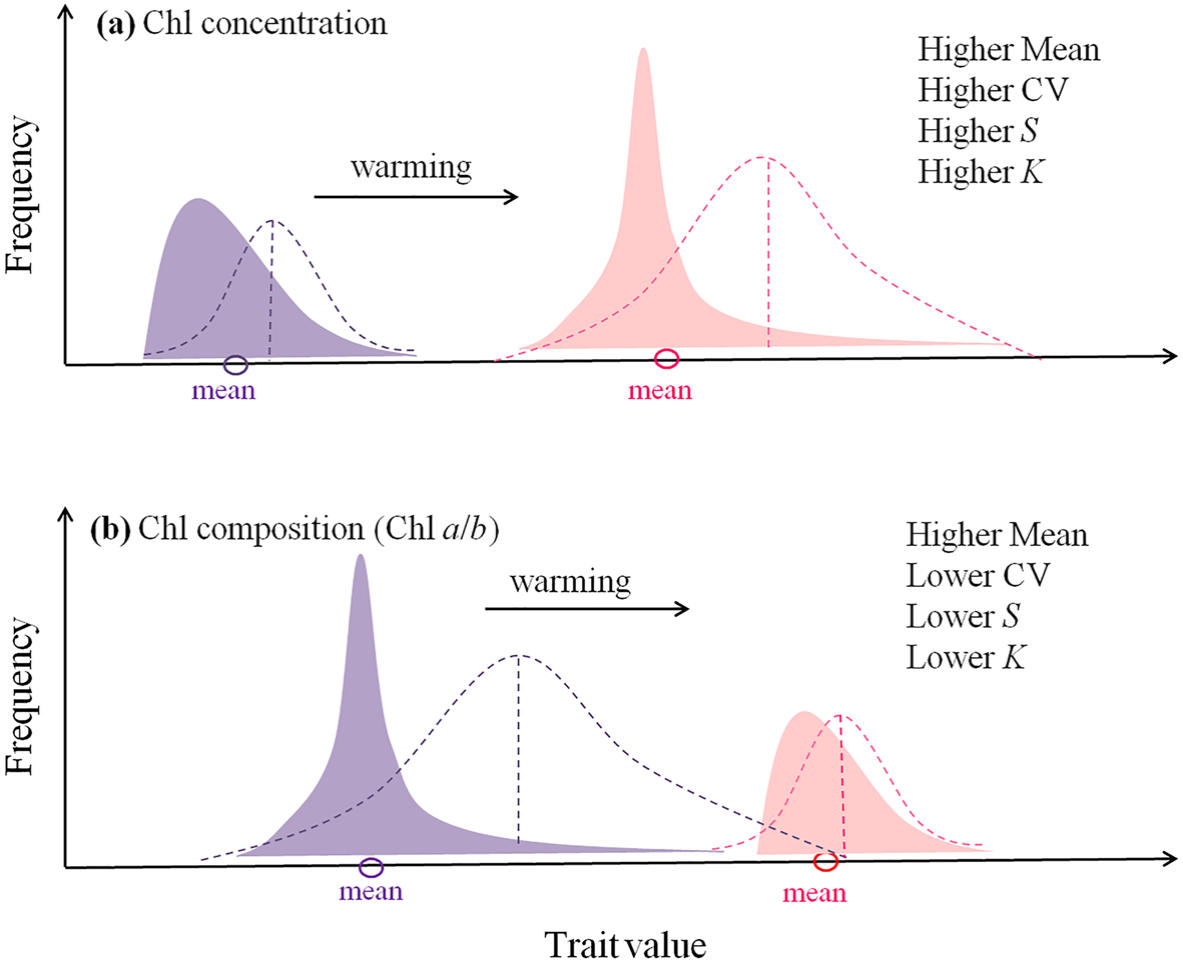
Theoretical sketches of distribution shifts for (**a**) chlorophyll concentration and (**b**) composition (Chl *a*/*b*) under global warming. Purple curves denote the current distributions, and pink ones represent the scenario under global warming. Dashed lines denote the normal distribution in the respective scenarios. It is supposed that the distribution of chlorophyll concentration will shift toward higher mean, CV, *S* and *K* values*,* while Chl *a*/*b* shifts toward higher mean but lower CV, *S* and *K* values under warming. *Chl* chlorophyll, *CV* coefficient of variation, *S* skewness, *K* kurtosis.

The CV represents the discrete degree of trait values, that is, the size of the trait space (Fig. 6b; Supplementary Note S1); *S* and *K* are generally used to describe the shape of trait distribution (Fig. 6c,d, Supplementary Note S1). Environment filtering can force a trait to deviate from the original distribution, with characteristics of smaller CV and larger *S* and *K* values^16,17^. Partly consistent with our hypothesis, MAT significantly exerted positive effects on the total concentration of CV, *S*, and *K*, but weaker negative effects on the three values of Chl *a*/*b* (Fig. 6a). That is, the distributions of Chl concentration and composition shifted in opposite directions under global warming: Chl concentration was distributed in a broader but more differential way (Fig. 7a), while Chl *a*/*b* was distributed in a narrower but more uniform way (Fig. 7b).

The trait distributional shift under warming is possibly caused by the relative role of species turnover and intraspecific variation (due to plasticity and/or heritable differentiation)^25^. For Chl concentration and composition, very weak phylogenetic signals were found in three plateaus (Supplementary Table S2), indicating the phenotypic plasticity of Chl, which environments have influenced during the long-term evolution. However, plasticity and intraspecific variation are not the focus of the discussion. Because the species compositions were significantly different among the three plateaus: with only a few species overlapping (Supplementary Fig. S3), and the dominant species and co-existing species gradually varied along the 30 sites (Supplementary Table S3). Shifts in Chl distributions under warming may be interpreted mainly by the alternation of species composition.

For Chl concentration, a broader trait space (higher CV) and a more skewed distribution (higher *S* and *K*) under warming conditions indicate several new species that differ in functions (here refer to rare species with higher Chl concentration) appeared or increased. This contributed to the long tail of the curve and raised the average Chl concentration. At the same time, most of the other species converged at lower Chl concentrations; that is, Chl concentration undergoes more substantial differentiation and functional contrasting species co-exist under warming. The concentration of Chl is representative of plant growth rate and production ability. Its distribution shift may imply a possible trend of polarisation in functions: both acquisitive and conservative species occur simultaneously. This alteration in species composition indicates changing biotic interactions^26^. The co-existence of functional contrasting species allows individuals to avoid competition and enhance the exploitation of resources and niche^27,28^, which is of great importance in optimising community functions^28,29^. In desert and alpine regions, functional contrasting species with large inter-specific trait variations improve community multi-functionality and enable better resistance to climate change^17,30^.

**Figure 8:**
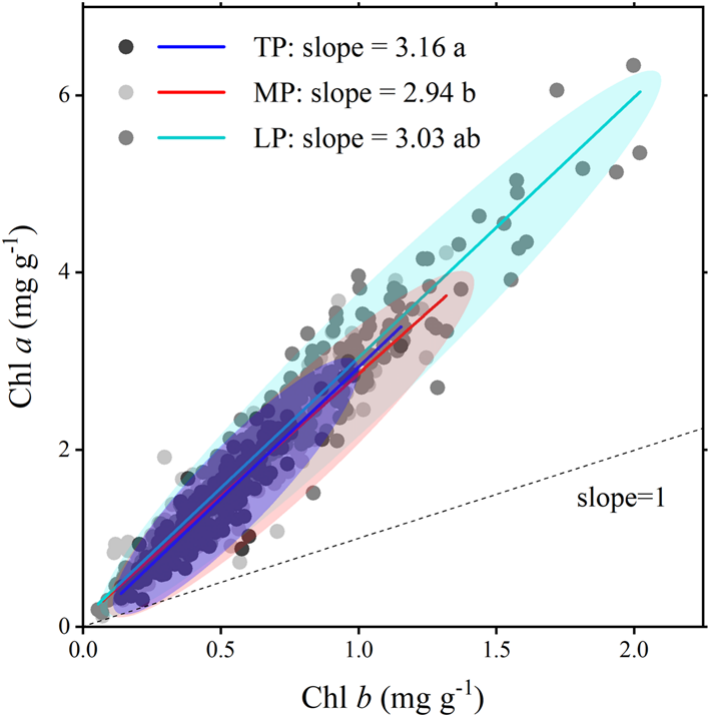
Standardised major axis regression of chlorophyll *a* to chlorophyll *b* in three plateaus. Slopes were given and compared among regions; different lowercase words denote significant (*p* < 0.05). *Chl* chlorophyll, *TP* Tibetan Plateau, *MP* Mongolian Plateau, *LP* Loess Plateau.

However, despite the shift in species composition, the distribution of Chl *a*/*b* only changed slightly compared to the Chl concentration under warming. The ratio of Chl *a* to Chl *b* represents the plant allocation to RC and LHC in PS and the efficiency trade-offs between light capture and light conversion^6,7^. This ratio is characteristic of conservatism which is mainly manifested in the following aspects: (1) Chl *a*/*b* is independent of Chl concentration (orthogonal relationship of the two; Supplementary Fig. S2); (2) Chl *a*/*b* distributed more converged with higher *K* and lower CV (Supplementary Table S1); (3) relative fixed allometric relationships were found between Chl *a* and Chl *b* (beside TP; Fig. 8). Plants may adjust their RC and LHC allocation to a common ratio of 3:1 despite large variations in light availability or Chl concentration, which has also been confirmed by a study from forests^14^. Considering that RC is costlier than LHC, plants tend to sustain the Chl *a*/*b* as low as possible unless there is a functional imbalance caused by environmental stress such as warming^9,31^.

Consequently, the shift in species composition may not be reflected by the conservative distribution of Chl composition. However, there is still a significant opposing trend for Chl *a*/*b*, indicating a highly uniform rather than a differential distribution of Chl composition under warming. The effect size is relatively weak, indicating that the shift in Chl composition may be slower or slighter than the Chl concentration under the same warming conditions and may not be suitable for prediction.

The effect of warming on community composition is a persistent concern, which will have severe consequences on community and ecosystem functions and processes. The significant reported effects of warming on grassland community structure include alterations in species coverage and important value^32^, reduction of dominant species and species richness^33^, conversion among functional groups^34^, and distribution shifts of phylogeny and function from divergence to randomness^35^. The conclusions vary depending on the regions, biomes, and succession stages of the community. For example, evidence from the tundra showed that warming typically supports many vascular species, shrubs, and fewer cryptogams^36^. A study in the northern Mongolian steppe found that locally restricted species are more sensitive to warming than more widespread species, based on their narrower niche breadth^37^. Fertile or early successional grasslands, composed of fast-growing and short-lived species, respond rapidly to climate warming^38^. Globally, warming has resulted in the migration of alpine vegetation toward higher latitudes and elevations, leading to the prevalence of thermophilic species^2,39,40^.

It is difficult to obtain a unified explanation for how the species respond to warming because species respond differently depending on their strategies, such as resource allocation, which will be influenced by warming through limitations on other functions or traits. For example, nitrogen (N) is a key component of Chl, photosynthetic enzymes, and other functional proteins. How the species allocate its leaf N to the photosynthetic apparatus under warming will greatly impact its growth, interaction with other species, and community structure. We examined the relationship between leaf N and Chl concentrations in the three plateaus. We found that a significantly higher proportion was allocated to Chl for the species in LP than in TP when leaf N increased by the same amount (Supplementary Figure S4). In TP, low temperatures may limit the metabolic and growth rates of species, and strong direct radiation may cause photodamage. In this case, the most important thing for a species is to survive rather than produce. The strategies adopted by species to enhance their persistence and tolerance are associated with increased resistance and protective proteins^41^, leading to competition for leaf N. In warmer places, the synthesis of leaf N will not be restricted, and species tend to allocate more leaf N to Chl to support production. Consequently, Chl concentration shifts under warming are possibly caused by changes in other traits. This can be checked through trait-trait relationships, indicating species-specific trade-offs between different functions.

Space-for-time substitutions use extant spatial patterns between biota and environmental factors to reveal the warm impacts on plants. However, this method was thought to overestimate the effect of warming because there are unavoidable covariations in geographical locations, soil resources, and climate factors, making it difficult to separate the warming effect independently^36,42^. In particular, species sensitivity to warming can be influenced by these covariation factors^36,37,43^. However, this is still a preferred method for predicting the long-term response of plants under warming because there are several ways to avoid the shortcomings of this method or modify the effect magnitude as much as possible^42,44^.

First, the study sites could be carefully selected with replications to avoid or minimise the coupling between temperature and potential confounding factors^42^. In this study, 30 sites were distributed mainly along the MAT gradient, which was almost independent of precipitation, soil resources, and aridity index (the orthogonal relationships showed between MAT and a suite of soil and climate factors; Supplementary Figure S5). This situation may be explained by the fact that the coupling of environmental factors was loosened at high elevation, so increasing temperature may not induce the additional effects of other environmental factors^45^. In addition, the 10 replicated sites within each plateau were distributed along the precipitation and soil nutrient gradients, with the availability of soil resources gradually decreasing from east to west (Supplementary Figure S5). Thus, additional effects of covariant factors may be offset in the entire 30 sites of grassland, and thus differences in geographical location are thought to translate into a temperature gradient. Although a weak negative correlation was observed between MAT and PAR, it was due to elevation change. However, despite the variability in elevation-climate relationships, it may be that at lower elevations, conditions tend to favour acquisitive species that can take advantage of high resource levels since higher temperatures stimulate microbial activity and increase resource availability and vice versa^44^. Consequently, species response and composition directions, shifting the Chl due to warming, should be reflected in this spatial transect.

Second, manipulative experiments or historical monitoring should be combined with space-for-time substitutions to draw a comprehensive conclusion^46^. We summarised recent artificial warming experiments conducted in these three plateaus to understand the warming effects on species composition and possible interactions with other factors. For example, an increase in temperature changed the important values and coverage of different species and functional groups in Inner Mongolia and Tibet grasslands. Still, a concurrent decrease in soil moisture hardly affected the community composition because the plants can morphologically spread their roots deeper to maintain water use^47,48^. Variations in precipitation induced by only warming influenced species phenology rather than composition^49,50^. However, some argued that the combined effects of increased temperature and precipitation exhibited strong spatial heterogeneity^51^, with more significant sensitivity of vegetation at higher elevations^52^. Concomitant N deposition may exert the same effect as warming on community structure but through different mechanisms, which affect asynchronous population dynamics^33^ or migration and availability of soil elements^53^. In addition, from a 6-year experiment, nitrogen addition did not affect the abundance of major species and functional groups while exerting a more direct effect on ecosystem functioning^54^. Although some conclusions support weak coupling between the roles of temperature and that of other factors, we should be aware of the defects of a large spatial span. In the plateau regions, the interaction between temperature and radiation should be considered. Informative results from manipulative experiments should be integrated into space-for-time substitutions to modify the effect size and consider the response time lag to warming in the near future.

In conclusion, warming may cause the Chl concentration to shift toward a broader but more differential distribution (functional heterogeneity), while Chl*a*/*b* shifted toward a narrower but more uniform distribution (functional homogeneity). The shifts in the Chl distribution curve may be caused by phenotypic plasticity, alternation in species composition, and leaf N allocation patterns of species. The Chl composition is more conserved and insensitive to environmental change, which may not be suitable for use as a tool to predict grassland dynamics under climate change. At the regional level, Chl concentration and composition depend on local climate and limiting factors. Space-for-time substitutions are an informative method for estimating the long-term response of plants under warming. Still, the study sites should be carefully selected to minimise the additional effects of covariation factors, and conclusions should be drawn at the base of artificial warming experiments and historical monitors.

## Methods

### Study area and site design

Northern Hemisphere grasslands are mainly distributed in the Eurasian continent. We designed 30 sampling sites without human disturbance at the core of the Eurasian steppe, which included three plateaus (Fig. 1a): TP, MP, and LP. Ten sites were set on each plateau, and contained three grassland types: meadow, typical grassland, and desert grassland from east to west. Large horizontal and vertical geographic gradients were involved with longitude 80.15–123.51° E, latitude 31.38–45.11° N, and altitude 144–4938 m. The sites were believed to represent northern hemispheric grasslands and suitable for the comprehensive study of Chl adaptation mechanisms.

The three plateaus are key vulnerable regions that have attracted scientific attention due to climate change. More importantly, the MAT differences of the three plateaus form a natural temperature gradient of *c*. ± 5 °C, with TP ‘ − 5 °C’, MP median, and LP ‘ + 5 °C’ (Fig. 1a, b). Despite the temperature gradient, the different spatial locations hardly affect Chl concentration among functional groups within each plateau (Supplementary Table S4). We wanted to track how grass Chl will adapt to current global warming trends using this large spatial MAT gradient. “Space-for-time substitutions” used multiple sites across an environmental gradient to predict a temporal trajectory in ecological change, which was assumed to be related to changes across the gradient^55^. We simulated temperature reciprocal transplants to take advantage of the MAT gradients, similar to transplanting the communities of TP (− 5 °C) to MP (0 °C) and then taking the new MP communities to LP (+ 5 °C) to identify changes in species pigments and reveal grassland production under global warming. Undoubtedly, during the “transplant”, community successions may occur and lead to different species composition because species have reduced fitness at the margin of their distribution range or complete loss fitness when transplanted beyond their current environments^56^. This phenomenon in “space-for-time substitutions” may occur in nature if the warming continues for a long time. In addition, due to the large span, space-for-time substitutions may provide additional variables (such as differences in physical, chemical, and ecological characteristics), which accumulate and interact with other covariant environmental variables. Nonetheless, these indirect effects are less pronounced on such a large gradient compared to direct climate effects^55^. Hence, the sites along the spatial MAT gradient we designed could estimate long-term warming within the systems (at least the response direction), based on the premise of the negligible effects of small-scale differences.

In addition to the significant temperature gradient among the plateaus, distinctive climate and resource conditions existed in each plateau. The three plateaus were characterised by strong radiation (Fig. 1e) but differed in limiting environmental factors. Specifically, temperature (Fig. 1b), precipitation (Fig. 1c), and soil N (Fig. 1d) were limited to TP, MP, and LP, respectively. Coincidentally, these factors all play essential roles in vegetation productivity, so we wanted to test whether the limiting factors could influence Chl concentration and composition at regional scales.

### Field sampling

Field sampling was conducted in July and August 2018 (growth peak for the northern grasslands). Because dominant species do not really represent all the species in natural communities, we collected all the different species that accessible within a 1 km radius (including trees, shrubs, and herbs) at each site. They were thought to be more accurate representatives of the whole species in these natural grassland communities. Overall, 475 species (belonging to 264 genera of 72 families) were surveyed and identified at 30 sites (125 species for TP, 177 species for MP, 192 species for LP, and a few overlapping species among the three plateaus). Three individuals were collected as replications for each species, and healthy and fully expanded leaves were chosen for Chl measurements. In addition, soil from the 0 to 10 cm layer was sampled randomly, with eight replications for each site. After drying naturally, removing the roots, and sieving through a 2 mm mesh, the soil samples were ground to powder using a ball mill (Mixer Mill MM400, Retsch GmbH, Haan, Germany) and stored at 4 °C for the determination of total N and total P.

### Measurement of chlorophyll concentrations

The Chl concentration (Chl *a*, Chl *b*, and Chl *a* + *b*) and composition (Chl *a*/*b*) of all 475 species were analysed, with three replications for each species. Fresh leaves (0.1 g) were weighed and extracted using 95% ethanol for three times, and the extraction solutions were combined, filtered and adjusted to a constant volume of 50 mL. Measurements were conducted using a spectrophotometer (UV-1700pharmaSpec UV–Vis spectrophotometer, Shimadzu Corporation, Kyoto, Japan). According to the Lambert–Beer law, the relationships between the Chl solution and optical density are.

1 $$ {\text{D}}_{{{665}}} = 83.31\;{\text{C}}_{{\text{a}}} + {18}{\text{.60}}\;{\text{C}}_{{\text{b}}} $$

2 $$ {\text{D}}_{{{649}}} = {24}.{\text{54 C}}_{{\text{a}}} + {44}.{\text{24 C}}_{{\text{b}}} $$

3 $$ {\text{G}} = {\text{C}}_{{\text{a}}} + {\text{C}}_{{\text{b}}} $$

where D_665_ and D_649_ are the optical densities of the Chl solution at wavelengths of 665 and 649 nm, respectively. C_a_ and C_b_ are the concentrations of Chl *a* and Chl *b* (g L^−1^), respectively. The coefficients 83.31 and 18.60 are the specific absorptions of Chl *a* and Chl *b* at a wavelength of 665 nm, while 24.54 and 44.24 are the specific absorption values at a wavelength of 649 nm. The concentrations of Chl *a*, Chl *b*, and total Chl (mg g^−1^ fresh mass), along with Chl composition, can consequently be calculated as^14,15^:

4 $$ {\text{Chl}}\,a\;\left( {{\text{mg g}}^{{ - {1}}} } \right) = {\text{C}}_{{\text{a}}} \times { 5}0/\left( {{1}000 \times 0.{1}} \right) $$

5 $$ {\text{Chl}}\,b\;\left( {{\text{mg g}}^{{ - {1}}} } \right) = {\text{C}}_{{\text{b}}} \times {5}0/\left( {{1}000 \times 0.{1}} \right) $$

6 $$ {\text{Total}}\;{\text{Chl}}\;{\text{concentration}} = {\text{Chl}}\,a + {\text{Chl}}\,b $$

7 $$ {\text{Chl}}\;{\text{composition}} = {\text{Chl}}\,a/{\text{Chl}}\,b $$

### Measurement of total N and P in soil

Soil samples (150 mg) were weighed, and the total N concentration was determined using a Vario MAX CN elemental analyser (Elementar Analysensysteme GmbH, Langenselbold, HE, Germany). Soil samples (0.05 g) were soaked in 6 mL nitric acid (HNO_3_) and 3 mL hydrofluoric acid (HF) overnight and boiled to invoke digestion using a Mars X press Microwave Digestion System (CEM Corporation, Matthews, NC, USA). Next, 0.5 mL perchloric acid (HCLO_4_) was added to remove the acid, and then the solutions were cooled and adjusted to 15 mL to measure total P^57^. Determinations were conducted using an inductively coupled plasma optical emission spectrometer (Optima 5300 DV, Perkin Elmer, Waltham, MA, USA). After determining total N and P, the soil N:P and averages for each site were calculated.

### Climate data

Data for PAR were obtained from ‘A dataset of reconstructed photosynthetically active radiation in China (1961–2014)’^58^; MAT and MAP data were obtained from the National Meteorological Information Center of China.

### Data analysis

All species were used for frequency distribution analysis with IBM SPSS Statistics 19.0 (SPSS Inc., Chicago, IL, USA). One-way ANOVA was used to test Chl averages among plateaus. Multiple comparison tests were performed using the least significant difference method (IBM SPSS Statistics 19.0, SPSS Inc., Chicago, IL, USA). The contributions to Chl concentration and composition variances were analysed using a nested-ANOVA by Minitab 18.1 (Minitab Inc., Pennsylvania State University, PA, USA). Standardised major axis analysis and slope comparisons were conducted using the ‘smart’ package of R (version 3.5.2, R Foundation for Statistical Computing) to reveal the allocations of Chl *a* and Chl *b* in the three plateaus. Principal component analysis, redundancy, and phylogenetic analyses were also performed using R to show the relationships among Chl indicators and between environments and Chl and the influences of phylogenesis on Chl. The figures were created using OriginPro 2018C (OriginLab Corp., Northampton, MA, USA).

### Statement for permission

No permits were required for this study in The People’s Republic of China because this study was conducted on public land, and neither natural reserves nor protected species were involved. The collection and handling of species materials were conducted at the research sites of the Chinese Ecosystem Research Network (CERN). They were in accordance with the institutional regulations of CERN and the Chinese Academy of Sciences.

## Supplementary Information

Supplementary Information.

## Data Availability

The primary data used in the paper are available in the Dryad dataset: https://doi.org/10.5061/dryad.n02v6wwvc.
