# Supplementary material for: Opposing shifts in distributions of chlorophyll concentration and composition in grassland under warming
Source: Sci Rep. 2021 Aug 3;11:15736. doi: 10.1038/s41598-021-95281-3 (PMC8333091; doi:10.1038/s41598-021-95281-3)
Supplement: Supplementary file 1 — Supplementary Information. [file 41598_2021_95281_MOESM1_ESM.docx]

|  | Chl *a* (mg g^-1^) | | | |  | Chl *b* (mg g^-1^) | | | |  | Chl *a*+*b* (mg g^-1^) | | | |  | Chl *a*/*b* | | | |
| --- | --- | --- | --- | --- | --- | --- | --- | --- | --- | --- | --- | --- | --- | --- | --- | --- | --- | --- | --- |
|  | TP | MP | LP | All |  | TP | MP | LP | All |  | TP | MP | LP | All |  | TP | MP | LP | All |
| Mean | 1.28 | 1.50 | 1.91 | 1.62 |  | 0.44 | 0.51 | 0.62 | 0.54 |  | 1.72 | 2.01 | 2.53 | 2.16 |  | 2.89 | 3.02 | 3.13 | 3.03 |
| CV | 0.41 | 0.44 | 0.49 | 0.50 |  | 0.39 | 0.45 | 0.50 | 0.49 |  | 0.40 | 0.44 | 0.49 | 0.49 |  | 0.16 | 0.22 | 0.11 | 0.17 |
| Skewness | 0.79 | 0.87 | 1.21 | 1.41 |  | 0.87 | 0.65 | 1.38 | 1.48 |  | 0.81 | 0.81 | 1.23 | 1.43 |  | 0.17 | 3.59 | 0.32 | 2.78 |
| Kurtosis | 0.73 | 1.15 | 2.33 | 3.47 |  | 1.43 | 0.35 | 2.97 | 4.06 |  | 0.93 | 0.88 | 2.42 | 3.58 |  | 2.86 | 22.42 | 0.71 | 24.11 |

**Table S1** Eigenvalues of frequency distribution of chlorophyll concentration and composition.

Chl: chlorophyll, TP: Tibetan Plateau, MP: Mongolian Plateau, LP: Loess Plateau, All: all species along northern hemispheric grassland transects, CV: coefficient of variation.

**Table S2** Phylogenetic signals of chlorophyll concentration and composition.

| Region | Chl *a* | |  | Chl *b* | |  | Chl *a*+*b* | |  | Chl *a*/*b* | |
| --- | --- | --- | --- | --- | --- | --- | --- | --- | --- | --- | --- |
|  | *K* | *p* |  | *K* | *p* |  | *K* | *p* |  | *K* | *p* |
| TP | 0.051 | 0.070 |  | 0.047 | 0.206 |  | 0.049 | 0.107 |  | 0.096 | 0.021* |
| MP | 0.035 | 0.007** |  | 0.036 | 0.003** |  | 0.039 | 0.001** |  | 0.012 | 0.566 |
| LP | 0.002 | 0.901 |  | 0.002 | 0.909 |  | 0.002 | 0.909 |  | 0.020 | 0.041* |
| All | 0.002 | 0.931 |  | 0.002 | 0.935 |  | 0.002 | 0.936 |  | 0.028 | 0.001** |

Chl: chlorophyll, *K*: strength of phylogenetic signal, *p*: significance level (*p* < 0.01**, *P* < 0.05*). TP: Tibetan Plateau, MP: Mongolian Plateau, LP: Loess Plateau; All: all species in northern grasslands.

**Table S3** Spatial locations and the dominant species for 30 sites of the grassland transects.

| **Site** | **Latitude (°N)** | **Longitude (°E)** | **Altitude (m)** | **Dominant species** |
| --- | --- | --- | --- | --- |
| TP01 | 31.46 | 95.45 | 4104 | *Carex thibetica*,  *Kobresia pygmaea*  *Potentilla saundersiana* |
| TP02 | 31.85 | 93.53 | 4509 | *Carex thibetica*  *Kobresia pygmaea*  *Polygonum viviparum* |
| TP03 | 31.64 | 92.01 | 4587 | *Kobresia pygmaea*  *Potentilla saundersiana*  *Saussurea japonica* |
| TP04 | 31.38 | 90.74 | 4617 | *Stipa capillata*  *Carex thibetica*  *Astragalus adsurgens* |
| TP05 | 31.54 | 89.72 | 4588 | *Stipa capillata*  *Carex thibetica*  *Leontopodium leontopodioides*  *Potentilla bifurca* |
| TP06 | 31.87 | 87.82 | 4570 | *Stipa capillata*  *Carex thibetica*  *Stipa purpurea* |
| TP07 | 31.92 | 85.84 | 4938 | *Leontopodium leontopodioides*  *Carex thibetica*  *Stipa purpurea* |
| TP08 | 32.41 | 83.34 | 4578 | *Poa annua*  *Eragrostis pilosa*  *Astragalus membranaceus*  *Stipa capillata* |
| TP09 | 32.30 | 81.23 | 4558 | *Stipa capillata*  *Astragalus membranaceus*  *Carex thibetica*,  *Artemisia desertorum* |
| TP10 | 32.48 | 80.15 | 4328 | *Stipa tianschanica*  *Stipa capillata*  *Ajania fruticulosa* |
| MP01 | 44.59 | 123.51 | 144 | *Chloris virgata*  *Leymus chinensis*  *Phragmites australis* |
| MP02 | 44.52 | 121.04 | 269 | *Setaria viridis*  *Ephedra sinica*  *Agropyron cristatum* |
| MP03 | 45.11 | 120.33 | 660 | *Setaria viridis*  *Cleistogenes songorica*  *Leymus chinensis* |
| MP04 | 44.77 | 118.36 | 1019 | *Stipa capillata*  *Agropyron cristatum*  *Leymus chinensis*  *Carex korshinskyi* |
| MP05 | 44.26 | 116.52 | 1129 | *Chenopodium glaucum*  *Stipa capillata*  *Leymus chinensis*  *Salsola collina*  *Cleistogenes songorica* |
| MP06 | 43.55 | 116.67 | 1272 | *Stipa capillata*  *Achnatherum sibiricum*  *Leymus chinensis* |
| MP07 | 44.51 | 117.68 | 1024 | *Carex korshinskyi*  *Cleistogenes songorica*  *Anemarrhena asphodeloides*  *Leymus chinensis* |
| MP08 | 44.01 | 114.89 | 1101 | *Salsola collina*  *Allium polyrhizum*  *Leymus chinensis* |
| MP09 | 43.84 | 113.50 | 1022 | *Allium bidentatum*  *Carex korshinskyi*  *Tribulus terrester*  *Eragrostis pilosa* |
| MP10 | 43.63 | 112.15 | 955 | *Tribulus terrester*  *Allium bidentatum*  *Corispermum mongolicum*  *Salsola collina* |
| LP01 | 36.29 | 113.36 | 804 | *Leymus chinensis*  *Bothriochloa ischaemum*  *Lespedeza bicolor*  *Viola philippica*  *Carex lanceolata* |
| LP02 | 35.99 | 112.29 | 894 | *Carex lanceolata*  *Themeda japonica*  *Tripolium vulgare* |
| LP03 | 35.99 | 111.64 | 833 | *Poa annua*  *Artemisia argyi*  *Lespedeza bicolor* |
| LP04 | 36.07 | 110.18 | 966 | *Tripolium vulgare*  *Bothriochloa ischaemum*  *Wikstroemia chamaedaphne* |
| LP05 | 36.74 | 109.24 | 1268 | *Lespedeza daurica*  *Bothriochloa ischaemum*  *Tripolium vulgare* |
| LP06 | 36.93 | 107.92 | 1383 | *Cleistogenes songorica*  *Tripolium vulgare*  *Stipa capillata* |
| LP07 | 37.58 | 107.19 | 1535 | *Leymus chinensis*  *Carex lanceolata*  *Artemisia scoparia* |
| LP08 | 37.42 | 105.78 | 1293 | *Cleistogenes songorica*  *Reaumuria songarica*  *Alopecurus aequalis* |
| LP09 | 37.44 | 104.92 | 1378 | *Heteropappus altaicus*  *Cleistogenes squarrosa*  *Sarcozygium xanthoxylon* |
| LP10 | 37.46 | 104.44 | 1714 | *Reaumuria songarica*  *Suaeda glauca*  *Eragrostis pilosa*  *Kalidium foliatum* |

TP: Tibetan Plateau, MP: Mongolian Plateau, LP: Loess Plateau.

| Functional group | TP | MP | LP | ALL |
| --- | --- | --- | --- | --- |
| Tree | 1.74±0.27 | 2.23±1.10 | 2.80±1.25 **a** | 2.67±1.23 **a** |
| Shurb | 2.07±0.59 **AB** | 1.97±0.74 **B** | 2.62±1.29 **Aab** | 2.36±1.12 **ABb** |
| Herb | 1.70±0.67 **C** | 2.00±0.86 **B** | 2.47±1.22 **Ab** | 2.09±1.01 **Bc** |
| Annual | 1.86±0.90 **B** | 1.93±0.97 **B** | 2.56±1.30 **A** | 2.22±1.17 **B** |
| Biennial | 1.61±0.85 **B** | 1.98±0.46 **AB** | 2.67±1.44 **A** | 2.24±1.24 **AB** |
| Perennial | 1.74±0.65 **C** | 2.05±0.86 **B** | 2.53±1.21 **A** | 2.18±1.03 **B** |
| Legumes | 1.87±0.48 **Cb** | 2.17±0.78 **BCb** | 3.09±1.38 **Aa** | 2.46±1.14 **Bb** |
| Gramineae | 2.40±0.85 **BCa** | 2.65±0.82 **BCa** | 3.08±1.38 **Aa** | 2.79±1.12 **ABa** |
| Compositae | 1.41±0.43 **Cc** | 1.73±0.65 **BCc** | 2.23±1.08 **Ab** | 1.91±0.93 **Bc** |
| Others | 1.67±0.64 **Cb** | 1.83±0.82 **Cc** | 2.40±1.15 **Ab** | 2.03±0.99 **Bc** |

**Table S4** One-way ANOVA for Chl *a*+*b* concentration (mg g^-1^) of different functional groups in three plateaus and entire grasslands.

Mean ± standard deviations are shown. Different lowercase letters denote significant differences among functional groups within each plateau, different capital letters denote significant differences among plateaus (*p* < 0.05). TP: Tibetan Plateau, MP: Mongolian Plateau, LP: Loess Plateau, All: all species along northern hemispheric grassland transects.

**Note S1** A brief introduction to the trait distribution indicators.

Trait distributions are usually visualised and quantified using frequency histograms and curves fitted from the histograms. Four eigenvalues of the curve are commonly focused on to understand the properties of a distribution. They are **mean**, **variance**, **skewness**, and **kurtosis**, all of which are of great importance in an ecological sense.

The **mean** of a trait implies the “trait value” of optimal adaptation for species under given environments, and means have been used in many trait studies at the individual or species level. The mean can be calculated as (Equation 1):

$\text{Mean}\text{ = }\frac{\text{1}}{\text{n}}\sum_{\text{i}}^{\text{n}} x_{i}$ (1)

where *x*_i_ is the trait value of individual “i” in a sample, and n is the sample size, that is, individual numbers.

**Variance** equals the square of the standard deviation (SD) and is used to describe statistical dispersion (Equation 2). However, when there are large differences in measure scales or dimensions between two groups of data sets, the normalised coefficient of variation (CV) is better used, as in the current study (Equation 3):

$Variance ={SD}^{2}= \frac{1}{n}\sum_{i}^{n} {(x_{i}-Mean)}^{2}$ (2)

$CV= \frac{SD}{Mean}= \frac{\sqrt{Variance}}{Mean}=\frac{\sqrt{\frac{1}{n}\sum_{i}^{n} {(x_{i}-Mean)}^{2}}}{Mean}$ (3)

where SD is the standard deviation, *x*_i_ is the trait value of individual “i” in a sample, and n is the sample size. Variance and CV reflect the variation in ecology, that is, the dispersion of the trait. A larger CV can be considered to represent a wider range of trait distribution or broader trait space, and *vice versa* (Fig. S1a).

**Skewness** (*S*) and **kurtosis** (*K*) are descriptions of the shapes of the distribution curves. The skewness represents the asymmetry of the distributions (Equation 4): a higher *S* with a right-/left-hand long tail indicates that the majority of trait values are distributed in one extreme and the minority in the other extreme (i.e. the long tail). In addition, positive or negative *S* values indicate the directions of the “majority” and “minority” (Fig. S1b). Kurtosis is the peakiness of the distributional curves (Equation 5), and a higher *K* is a symbol of converged distribution (Fig. S1c).

$Skewness= \frac{\frac{1}{n}\sum_{i}^{n} {(x_{i}-Mean)}^{3}}{{Variance}^{3/2}}= \frac{\frac{1}{n}\sum_{i}^{n} {(x_{i}-Mean)}^{3}}{\left[ \frac{1}{n}\sum_{i}^{n} {(x_{i}-Mean)}^{2} \right]^{3/2}}$ (4)

$Kurtosis= \frac{\frac{1}{n}\sum_{i}^{n} {(x_{i}-Mean)}^{4}}{{Variance}^{2}}= \frac{\frac{1}{n}\sum_{i}^{n} {(x_{i}-Mean)}^{4}}{\left[ \frac{1}{n}\sum_{i}^{n} {(x_{i}-Mean)}^{2} \right]^{2}}$ (5)

It is easily found from the mathematical formula that similarities exist between *S* and *K*. Moreover, they were proven to be significantly relevant to each other. The skewness–kurtosis relationship confirmed that distribution with higher skewness would also be more peaked with a large probability, indicating a converged trait distribution or decreased trait diversity under environmental filtering.

Means, CV, *S*, and *K* values within a given community change with the environment, which further suggests feedback between environments and functional trait distributions. The Traits Drivers Theory provides a framework for understanding how individual traits affect community patterns and is applicable across different geographic gradients and temporal and spatial scales. It was argued that the shape and dynamics of trait distributions could be linked to the fundamental drivers of community assembly and the response to future climate change. Consequently, research should focus on trait distribution and dynamics across broad geographic and climatic gradients and how these distributions influence ecosystem function.

**

Figure S1** Sketches of alterations in coefficient of variation (CV), skewness (*S*), and kurtosis (*K*) of trait distribution within a community under environmental filtering. (a) CV alternations, (b) *S* alternations, and (c) *K* alternations. Dashed lines: original distributions; solid lines: shifting distribution under environmental filtering.

**

Figure S2** Principal component analysis (PCA) of chlorophyll concentration and composition. Dots: species samples, red diamonds: chlorophyll (Chl) *a*, *b*, *a*+*b* and *a*/*b*.


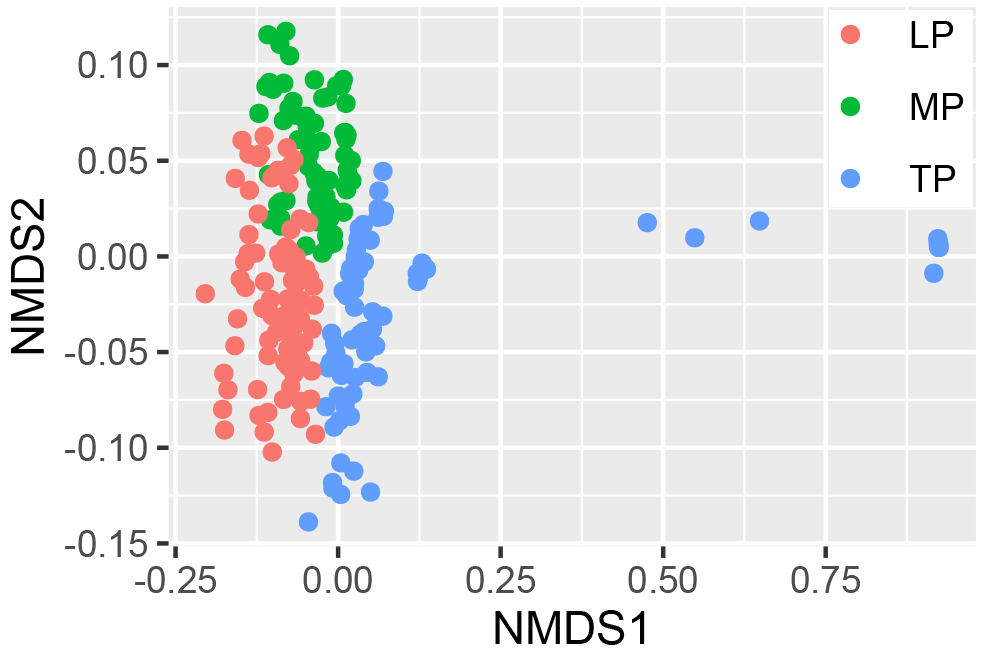
**Figure S3** Dissimilarity of Species composition in the three grassland plateaus demonstrated by non-metric multidimensional scaling (NMDS). TP: Tibetan Plateau, MP: Mongolian Plateau, LP: Loess Plateau.


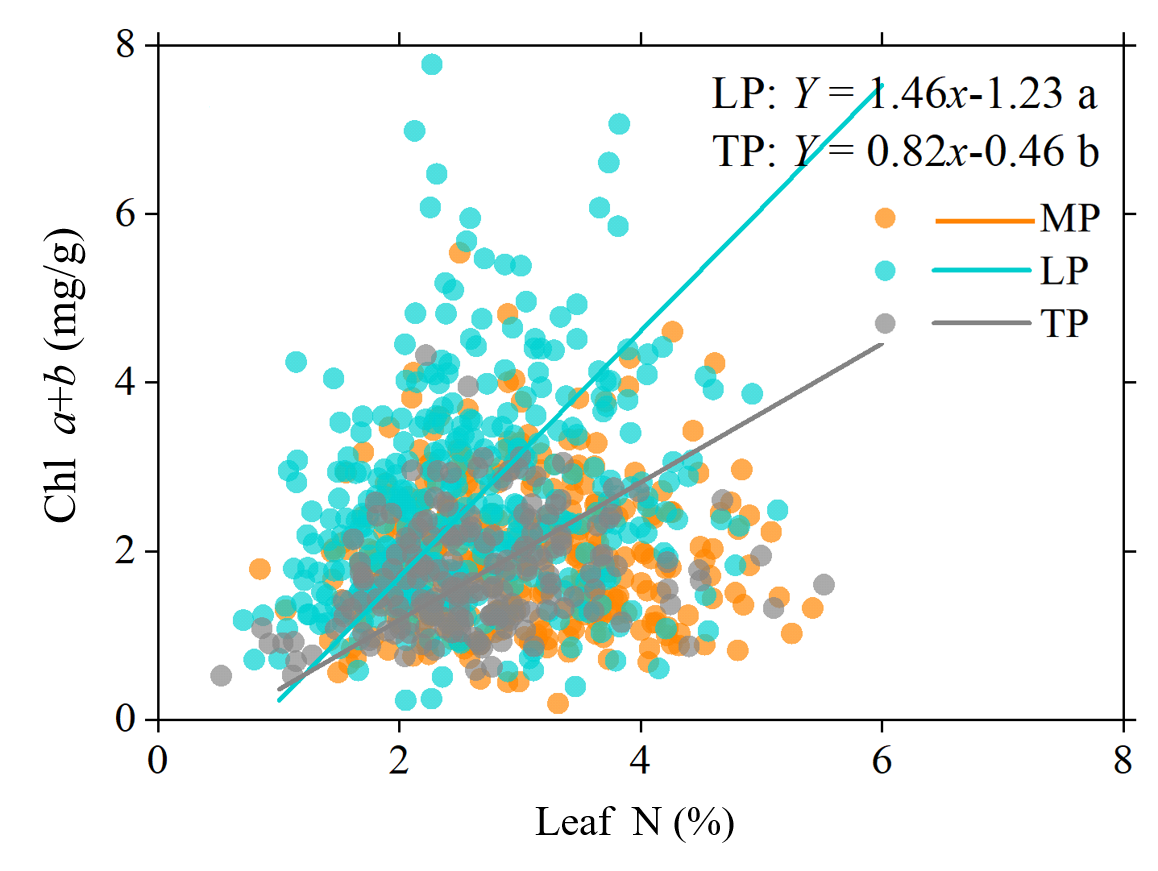
**Figure S4** Standardized major axis (SMA) regression of chlorophyll concentration (Chl *a*+*b*) to leaf N in three plateaus. Lines denote significant linear relationships (*p* < 0.05). Fitting functions are given only for significant linear relationships. Different lowercase letters behind functions indicate a significant difference between SMA slopes (*p* < 0.05). TP: Tibet Plateau, MP: Mongolia Plateau, LP: Loess Plateau.

**
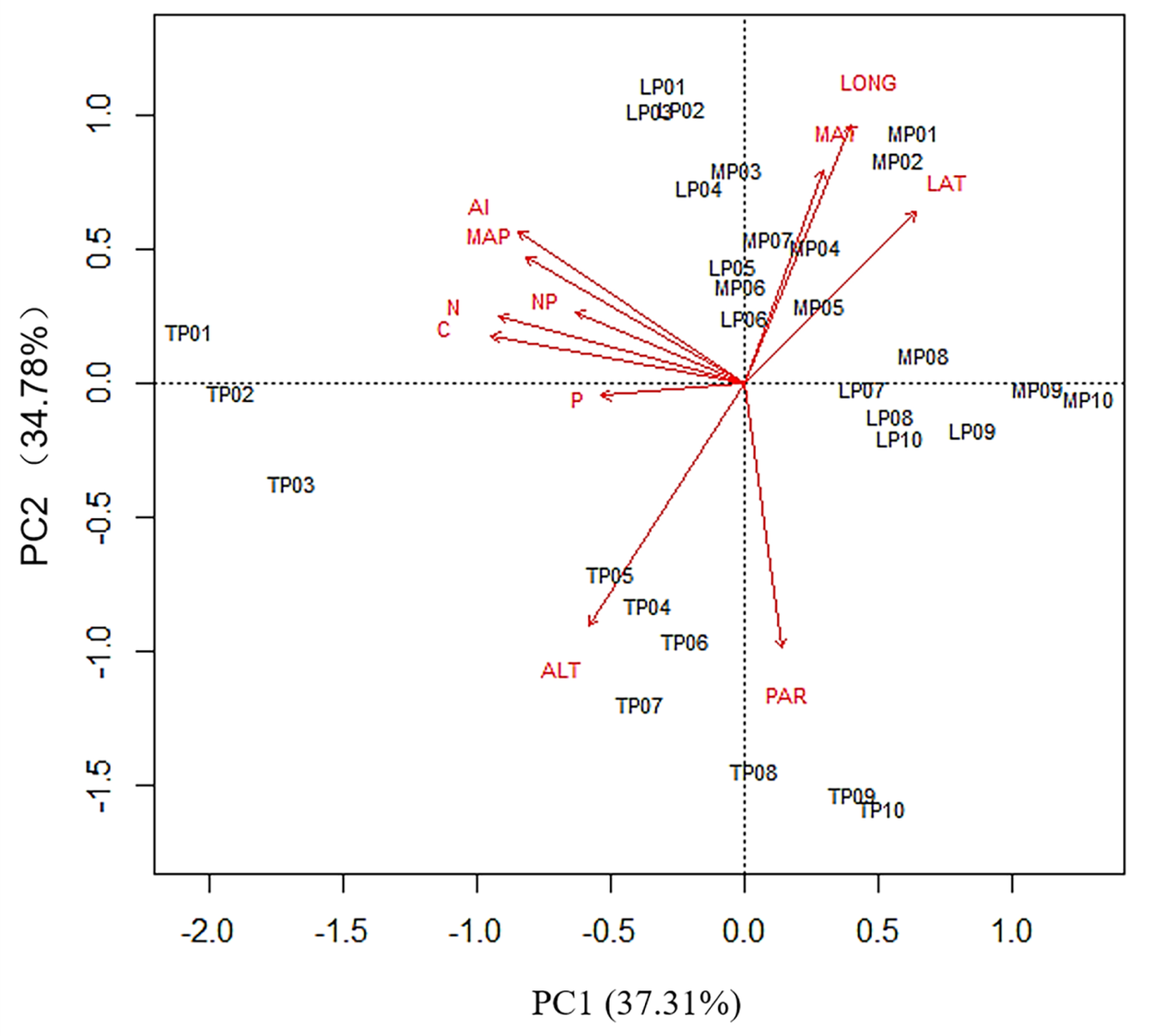
 Figure S5** Principal Component Analysis (PCA) of geographical and environmental variables of 30 study sites in the grassland transects. LONG: longitude, LAT: latitude, ALT: altitude, PAR: photosynthetically active radiation, MAP: mean annual precipitation, MAT: mean annual temperature, NP: the ratio of soil nitrogen to phosphorus, C: soil total carbon, N: soil total nitrogen, P: soil total phosphorus, and AI: aridity index. TP: Tibet Plateau, MP: Mongolia Plateau, LP: Loess Plateau.
